# Gene Editing for the Treatment of Primary Immunodeficiency Diseases

**DOI:** 10.1089/hum.2020.185

**Published:** 2020-10-16

**Authors:** Rajeev Rai, Adrian J. Thrasher, Alessia Cavazza

**Affiliations:** Infection, Immunity and Inflammation Research and Teaching Department, Great Ormond Street Institute of Child Health, University College London, London, United Kingdom

**Keywords:** gene editing, primary immunodeficiency diseases, blood disorders

## Abstract

With conventional treatments for primary immunodeficiency diseases (PIDs), such as allogeneic stem cell transplantation or autologous gene therapy, still facing important challenges, the rapid development of genome editing technologies to more accurately correct the mutations underlying the onset of genetic disorders has provided a new alternative, yet promising platform for the treatment of such diseases. The prospect of a more efficient and specific therapeutic tool has pushed many researchers to apply these editing tools to correct genetic, phenotypic, and functional defects of numerous devastating PIDs with extremely promising results to date. Despite these achievements, lingering concerns about the safety and efficacy of genome editing are currently being addressed in preclinical studies. This review summarizes the progress made toward the development of gene editing technologies to treat PIDs and the optimizations that still need to be implemented to turn genome editing into a next-generation treatment for rare monogenic life-threatening disorders.

## Introduction

Primary immunodeficiency diseases (PIDs) constitute a heterogeneous group of rare genetic disorders impairing the development, regulation, and function of the immune system. Increased susceptibility toward infections, autoimmunity, and inflammatory complications as well as risk of developing malignancies are major clinical manifestations observed in individuals affected by PIDs.^1^ The advancement in DNA sequencing technologies and genetic testing have now led to the identification of >300 gene mutations causing different forms of PID.^2^ Furthermore, distinct mutations in the same PID-related gene can lead to very different clinical and immunological disease phenotypes, increasing the complexity and heterogeneity of this group of disorders. Although the majority of PIDs are inherited, with an incidence rate approximately between 1 in 5,000 to 1 × 10^6^ live birth, acquired forms of the disease have been also described.^3^

The management of many PIDs is based on preventing infection through prophylaxis and where necessary anti-inflammatory medication.^4^ While the above treatments may provide adequate temporary support, allogeneic hematopoietic stem and progenitor cell (HSPC) transplantation has proven a definitive cure for many PIDs with a hematopoietic cell background. Unfortunately, the limited availability of Human Leukocyte Antigen (HLA)-matched donors poses a constraint for many patients, and although transplantation using HLA-mismatched donors is increasingly successful, it comes with significant risks, including graft versus host disease and graft rejection leading to incomplete immune cell reconstitution and higher risks of mortality and long-term morbidity. Autologous gene therapy provides an attractive option by genetically correcting the patient’s own HSPCs through the use of viral vectors.^5^ By improving safety features of viral vectors, current phase I/II gene therapy trials have been applied to the treatment of various PIDs, including adenosine deaminase-severe combined immunodeficiency (ADA-SCID), X linked-SCID (SCID-X1), chronic granulomatous disease (CGD) and Wiskott–Aldrich syndrome (WAS) with largely successful clinical outcomes.^6–10^ Despite this, longterm safety/efficacy follow-up studies are still required for most of these gene therapy products to be approved as medicinal drugs,^11^ as they come with some limitations, including the semirandom integration pattern of viral vectors and often unregulated transgene expression in transduced cells.^12^

Precise targeting by means of gene editing has recently emerged as an alternative technology to overcome the limitations of conventional gene therapy. Engineered endonucleases that introduce double-strand breaks (DSBs) at specific sequences in the genome offer much more control over viral vector integration. Moreover, the site-specific correction of the disease-causing mutant DNA *in situ* ensures that a physiologically regulated expression of the correct gene is more likely to be preserved in edited cells. In this review, we will illustrate the different types of gene editing platforms that have been developed so far and how these technologies have been utilized as therapeutic applications to treat severe forms of PID before, focusing on key challenges and mandatory requirements that gene editing needs to fulfill before translation into a cell therapeutic medicine for routine clinical application.

## Gene Editing Tools

**Figure 1:**
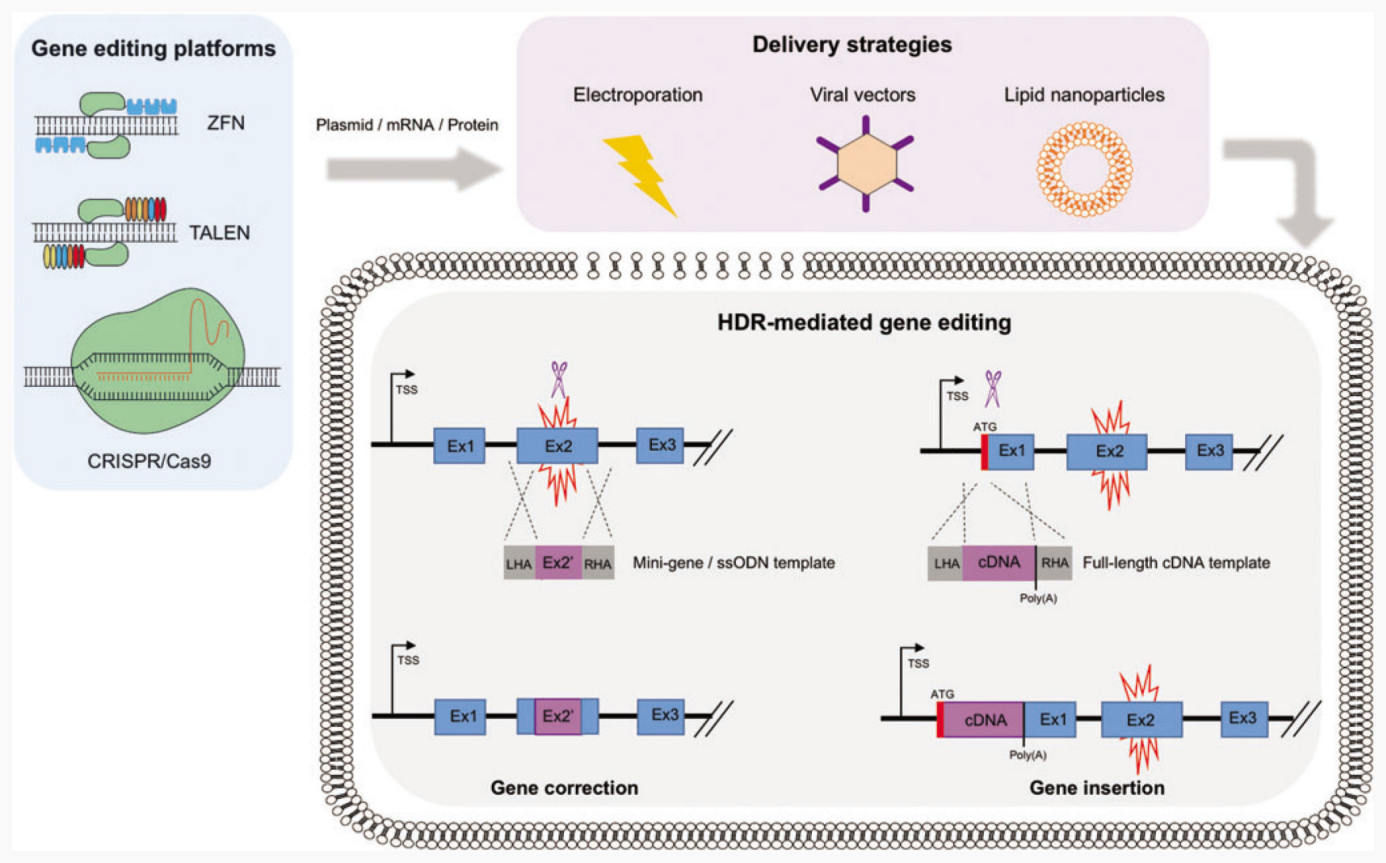
HDR-mediated genome editing strategies to achieve physiological expression of the correct gene. Gene editing platforms, such as ZFNs, TALENs, and CRISPR/Cas9, are delivered to the target cell in either a plasmid, RNA, and/or protein format (*blue box*). Strategies to introduce these reagents into the cells include electroporation, viral vectors, and lipid nanoparticles (*purple box*). Once the endonucleases reach the cell nucleus, upon binding to the DNA, they induce double-strand breaks (*blue* scissors) at specific sites. Two major HDR-based strategies can be implemented to revert a disease phenotype caused by a genetic mutation (*red* spark), while preserving endogenous regulation and wild-type levels of expression of the correct protein. In the case of gene correction, the delivery of a mini-gene or a ssODN donor template can replace small fragments of the mutated region or correct a few base-pair mutation. In the case of gene insertion, a wild-type cDNA donor template can be knocked-in close to or in frame with the translation start codon (ATG) of the mutated gene. Both strategies will result in the functional restoration of regulated and physiological protein expression, driven by endogenous transcriptional and post-transcriptional regulatory regions. CRISPR/Cas9, clustered regularly interspaced short palindromic repeats/associated Cas9 nuclease; HDR, homology directed repair; ssODN, single-strand oligo DNA; TALEN, transcription activator effector nuclease; ZFN, zinc finger nuclease. Color images are available online.

Over the past decade, there has been a global upsurge in the identification and discovery of novel gene editing tools. To date, in the context of PID research, three main platforms have been extensively utilized: Zinc finger nucleases (ZFNs), transcription activator effector nucleases (TALENs), and clustered regularly interspaced short palindromic repeats–associated Cas9 nuclease (CRISPR/ Cas9) (Fig. 1). These engineered nucleases consist of a site-specific DNA-binding domain and a nonspecific nuclease domain that cleaves the target DNA. ZNFs and TALENs contain zinc finger or TALE modules, respectively, as DNA-binding domains, which are linked to a dimerization-dependent *Fok*I endonuclease domain.^13^ The CRISPR/Cas9 platform relies on a DNA-binding guide RNA (gRNA), which is complementary to the target DNA sequence, and a Cas9 endonuclease.^14^ As a simple modification of the gRNA sequence is required to target different genetic loci, without the need of complex nuclease engineering as in the case of ZFNs and TALENs, CRISPR Cas9 has become a cost-effective, easy-to-use, and popular choice for genome editing among the scientifc community.

Although they differ in terms of target recognition, activity, and specificity, these platforms have been designed to create DSBs upon binding to the target site, triggering the activation of two main endogenous repair pathways: nonhomologous end joining (NHEJ) and homology-directed repair (HDR). Each of these pathways could be exploited for therapeutic purposes. The efficient but error-prone NHEJ pathway repairs the DSBs by ligating the two DNA strands directly without the need of a template, thus leading to the random insertion and/or deletion (indels) of nucleotides. The generated indels can abolish the expression of a protein or the function of a regulatory region and thus this repair pathway could be utilized to treat pathological dominantly active genetic elements. In contrast, HDR mediates the accurate repair of the cut by utilizing a DNA sequence homologous to the region flanking the DSB as a template. As this process results in the insertion of a correct DNA sequence, this pathway could be harnessed when treating those diseases, for which correcting or adding a genetic element may lead to a therapeutic benefit.

In the context of PIDs, HDR-mediated editing is the most frequently used repair pathway to correct loss-of-function mutations underlying the disease, by either sitespecific insertion of a gene or correction of patient-specific mutations (Fig. 1). Site-specific correction of diseasecausing mutations is the most elegant approach to repair a faulty gene responsible for a monogenic disorder and is particularly useful when tackling conditions in which a single or predominant mutation underlies the disease. However, the majority of PIDs are caused by mutations spanning across the genes, urging the tailoring of gene editing reagents for each individual patient. For this reason, integrating an entire corrective gene cassette to the desired locus through HDR-mediated site-specific gene insertion would functionally correct all disease-causing mutations with just one set of reagents, thus representing a more attractive approach that could be universally applied to all the patients. This strategy could be used to integrate a gene either into a specific “safe harbor,”^15^ or into its own genomic locus, allowing the preservation of its physiological expression. Although the latter strategy is specific for each single gene, it could be particularly amenable for diseases in which endogenous gene regulation is essential.

## Gene Editing Strategies to Treat PIDs

From the very first PID gene therapy clinical trial, successful treatment outcomes have been generated from the *ex vivo* modification of HSPCs. Since the isolation and autologous transplantation of HSPCs to patients is now relatively easily accomplished, PID clinical trials involving gene editing of HSPCs will likely follow the *ex vivo* route for the foreseeable future. Overall, the application of genome editing to HSPCs has shown substantial benefits toward functional gene correction for different types of PIDs. When applying genome editing with the aim to repair the functional and phenotypic defects of a disease, researchers not only need to choose which specific tool to utilize, but also the delivery route, dose, and timing of editing reagents. This is to ensure that high HDR efficiency, and negligible cytotoxic and off-target effects are achieved in the cell type of interest. Depending on the type and location of the mutation, researchers have shown that it is possible to fully recapitulate a physiological gene expression pattern and restore a functional immune response by HDR-mediated editing of the desired genomic locus in HSPCs. Evidence has also shown the ability of patient-derived edited cells to engraft the bone marrow and differentiate into various immune cells *in vivo.* Large-scale preclinical studies are underway to assess the long-term safety and efficacy of the gene editing products and to define the minimum level of targeted correction required to achieve a therapeutic benefit for each type of PID without significantly affecting lifelong haematopoiesis. In this study, we will review the current progresses in the genome editing field by highlighting the most promising strategies that have been put in place to correct PIDs.

### Severe combined immunodeficiency, X-linked

In 2005, Urnov *et al.* were among the first groups to demonstrate functional correction of a mutated *IL2RG* gene, which is responsible for SCID-X1, an X-linked recessive monogenic PID characterized by a complete block in the development of T and NK lymphocytes with dys-functional B cells.^16,17^ To edit *IL2RG,* Urnov et *al.* transfected K562 and T cells with ZFNs together with a donor plasmid carrying an exon 5 fragment of *IL2RG*, achieving a frequency of HDR-mediated repair of up to 5% in primary T cells.^17^ These experiments highlighted the limitations associated to the delivery of editing reagents through plasmid transfection in clinically relevant primary cells, where elevated cell toxicity and a negligible HDR frequency could be observed. To address these issues, Lombardo *et al*. utilized an integrase-defective lentiviral vector (IDLV) to package and deliver ZFN dimers and the donor template to target *IL2RG,* resulting in 0.2% of GFP-expressing HSPCs derived from healthy donors with minimum toxicity.^18^ To improve the overall *IL2RG*-targeting rates in HSPCs, Genovese *et al*. optimized the *ex vivo* culture conditions as well as timing and delivery route of the editing reagents.^19^ Through electroporation of ZFN mRNA 2 days after cell thawing, followed by IDLV transduction of an *IL2RG-GFP* donor template, an increase to up of 20% GFP-positive HSPCs was achieved,with a marked increase in the frequency and yield of GFP-positive targeted cells in primitive, long-term repopulating hematopoietic stem cells (HSCs). Recently, two additional studies have demonstrated a tremendous advancement in the correction of SCID-X1 by gene editing, using either the ZNF or CRISPR/Cas9 system coupled to the delivery of the donor template to HSPCs using an AAV6 vector.^20,21^ Both studies demonstrated that higher HDR-mediated editing can be achieved in HSPCs and HSCs population both *in vitro* and *in vivo* when cells are electroporated with a gRNA–Cas9 ribonucleoprotein (RNP) complex followed by transduction with an AAV6 donor template. Indeed, this strategy facilitated up to 40% of targeted integration in SCID-X1 HSPCs, fully restoring physiological gene expression and successfully reconstituting all hematopoietic lineages in immunodeficient mice transplanted with gene-corrected cells.^21^ SCID-X1 represents an ideal target for proof-of-principle gene editing studies, as the selective advantage that functionally corrected cells have over mutated ones in an SCID setting can compensate for the relatively low rate of HDR-mediated correction in HSPCs.^20^ Additional protocol optimizations may be required to increase the rates of gene correction in HSPCs and more primitive HSCs to revert the disease phenotype in PIDs, where such a strong selective advantage is missing.

### Chronic granulomatous disease

CGD is a group of life-threatening PID caused by mutations in any of the five protein subunits (gp91^phox^, p22^phox^, p40^phox^, p47^phox^, and p67^phox^) of the nicotinamide adenine dinucleotide phosphate oxidase (NADPH) enzyme complex. Apart from gp91^phox^, which is encoded by the *CYBB* gene located in the X chromosome and associated to the X-linked form of the disease, the remaining subunits are responsible for autosomal recessive CGD. Since the NADPH oxidase is essential for the production of reactive oxygen species and nuclear extracellular trap in phagocytes, CGD patients with nonfunctional NADPH oxidase are susceptible to severe infection and inflammation, including pneumonia and blood sepsis.^22^

Recent efforts to treat X-CGD through HSPC gene editing have seen the use of optimized ZFN and CRISPR/ Cas9 systems codelivered with either an AAV6 vector or single-strand oligo DNA (ssODN) as donor templates. By electroporating ZFN mRNA targeting the “safe harbor” AAVS1 locus, followed by transduction with an AAV6 containing a *CYBB* cDNA template, De Ravin *et al.* showed the restoration of gp91^phox^ expression in 15% of X-CGD HSPCs *in vitro,* whereas 4–11% gp91^phox^ + cells were detected in immunodeficient mice following transplantation of corrected cells. Despite relatively low HDR rates both *in vitro* and *in vivo*, >95% of the corrected cells reconstituted the oxidase activity and displayed similar gene expression level compared with wild-type controls.^23^ The same group later reported improved *in vitro* targeting rates of up to 31% in X-CGD HSPCs and HSCs when attempting site-specific correction of a point mutation in exon 7 of the CYBB gene by electroporation of the RNP complex with a ssODN donor template. Upon transplantation of manipulated HSPCs into immunodeficient mice, 15–20% of corrected cells persisted in transplanted animals for up to 5 months, with restored physiological expression of the gp91^phox^ in mature phagocytes and proper functionality of the NADPH oxidase.^24^ Despite these encouraging data, whether such gene correction is sufficient to alleviate the pathogenesis of CGD remains to be investigated, as corrected X-CGD cells do not have a selective advantage *in vivo*. It would also be interesting to conduct secondary and tertiary transplantation experiments with gene-edited HSPCs to verify whether endogenous physiological expression is maintained during long-term hematopoiesis *in vivo*.

Contrary to X-CGD, where multiple mutations are responsible for the onset of the disease, p47-CGD could be tackled with a gene correction, rather than gene insertion approach, as >80% of p47-CGD patients are homozygous for a two-nucleotide deletion (ΔGT) exon 2 of the *NCF1* gene, which interestingly, is also found in two *NCF1* pseudogenes, *NCF1A* and *NCF1C.* By electroporating ZFNs targeting *NCF1* exon 2 together with a AAV6 vector containing an exon 2 repair template for HDR-mediated correction, Merling *et al*. reported the replacement of the mutated exon in the *NCF1* locus as well as in both pseudogenes, restoring oxidase function in 6% of myeloid cells differentiated from p47-CGD-induced pluripotent stem cells (iPSCs).^25^ A similar approach was recently pursued by Klatt *et al.,* who employed a plasmid-based CRISPR/ Cas9 system to correct the ΔGT mutation in the *NCF1* locus of p47-CGD iPSCs, resulting in the complete re-constitution of phagocyte function without altering any sequences in the pseudogenes.^26^ Optimization of this mutation-specific approach and its application to clinically relevant HSPCs may provide a valid therapeutic alternative for patients suffering from p47-CGD.

### Wiskott–Aldrich syndrome

Compared with other PIDs, such as SCID-X1 and CGD, where the immune defect is restricted to either lymphocytes or granulocytes, WAS patients suffer from a complex functional defect in all mature hematopoietic cells, with the exception of red blood cells. This X-linked disease is caused by mutation in the *WAS* gene encoding the WAS protein (WASp), an actin cytoskeleton regulator. Clinical manifestation of WAS includes thrombocytopenia, recurrent infection, eczema, autoimmunity, and cancer.^27^ The first proof-of-concept study for the use of gene editing to tackle WAS was reported by Laskowski *et al.* in patient-derived iPSCs using a plasmid-based ZNF platform. Despite very low rates of targeted integration,protein expression level in edited iPSC-derived hematopoietic cells were comparable to wild-type controls, and functional correction of NK cells was demonstrated.^28^ By using ZFN and CRISPR/Cas9 plasmid-based systems, a later study compared the efficiency of integration of a GFP cassette in WAS intron 1 in K562 cells, achieving an HDR frequency of 5%.^29^ Despite encouraging early results, such reduced levels of targeted integration achieved in cell lines are unlikely to lead to a therapeutic correction of the defects associated with WAS when applied to patient-derived HSPCs. Our group has recently overcome this limitation by developing a highly specific and efficient CRISPR/Cas9 coupled to AAV6 editing system to insert a codon-optimized WAS cDNA in frame with its endogenous translation start codon in wild-type and WAS HSPCs.^30^ This strategy not only resulted in up to 60% of targeted integration in WAS HSPCs derived from various patients, but also significantly restored the functional and phenotypic defects in macrophages, platelets, T and B cells derived from corrected HSPCs, associated with relatively physiological expression of WASp.^30^ Primary and secondary transplantation of edited HSPCs into immuno-deficient mice revealed the preservation of their differentiation potential *in vivo,* with evidence of corrected WASp expression being preserved in long-term repopulating cells and lack of any noticeable toxicity. This proof-of-concept study provides a demonstration of the efficacy and the safety of a CRISPR/Cas9-based gene editing approach to treat WAS, setting the ground for an alternative, yet highly efficient, safe, and precise treatment for this disease.

### X-linked hyper-IgM syndrome

For some PID, especially those caused by mutations in genes that require a tightly regulated expression, introduction of the correct gene in its own locus by targeted gene editing could represent the strategy of choice. X-linked hyper-IgM syndrome (XHIM) is caused by mutations in the CD40 ligand (*CD40L*) gene. Expression of *CD40L* is finely tuned so that its protein is only upregulated on the surface of activated T cells, where it induces the immunoglobulin class switch upon binding to CD40 on B cells. Without effective antibody production, XHIM male infants are prone to recurrent infection and autoimmunity with reduced chance of survival.^31^ Although previous gene therapy attempts in an XHIM mouse model reconstituted adaptive immunity,^32^ the constitutive ectopic expression of CD40L from the viral promoter resulted in the abnormal proliferation of transduced T cells leading to the development of lymphomas in the experimental mice (in the apparent absence of insertional mutagenesis). Hubbard *et al.* tried to overcome this bottleneck by targeting a correct *CD40L* gene under the control of its endogenous promoter in primary T cells from three different XHIM donors using TALENs coupled with AAV6 as a donor template, reaching up to 30% of HDR-mediated targeted integration.^33^ Importantly, gene-edited cells recapitulated the wild-type T cell activation–resting kinetics both *in vitro* and *in vivo*, which is indicative of normal *CD40L* physiological restoration, and restored IgG class switching. Although autologous transplantation of *CD40L*-edited T cells is feasible for treating XHIM and could be used as a bridge therapy before curative HSC transplant, a sustained life-long clinical benefit probably requires correction of the mutated gene at the HSPCs level. Kohn’s group developed TALEN and CRISPR/Cas9-based platforms to correct the functional defects of XHIM in healthy donor HSPCs, reporting a relatively high gene integration rate in CRISPR/Cas9-edited cells and no aberrant multilineage differentiation both *in vitro* and *in vivo*.^34^ An average of 4% of edited cells persisted for more than 5 months after transplantation in immunodeficient mice, demonstrating correction of long-term repopulating HSCs, and more than 60% of the mice showed thymic reconstitution. The results obtained in primary T cells and HSPCs suggest that a small number of gene-corrected T cells may be sufficient to restore IgG class switching and ameliorate the disease phenotype. These promising data may provide a backbone for further work to address whether edited patient-derived XHIM-HSPCs can be differentiated into corrected T cells without the occurrence of any adverse event.

### Immune dysregulation, polyendocrinopathy, enteropathy, X-linked (IPEX)

Immune dysregulation, polyendocrinopathy, enteropathy, X-linked (IPEX) is another example of an X-linked monogenic PID, where the application of gene editing is desirable to preserve the cell-type-specific expression of the gene upon targeted integration. The disease is caused by mutations in the *FOXP3* gene, which is constitutively expressed in regulatory T cells (Treg) and transiently up-regulated in activated effector T cells (Teff). Impairment of both Treg and Teff functions underlies the onset of IPEX and patients present with many different autoimmune manifestations, such as type 1 diabetes, life-threatening enteropathy, eczema, and cytopenia.^35^ For the development of a lifelong *FOXP3* gene therapy using HSPCs, it is necessary to achieve constitutive expression of *FOXP3* in the Treg compartment without having *FOXP3* overexpression perturb the proliferation and function of HSPCs or Teff cells. To achieve such cell-type-specific expression, a recent study has described a one-size-fits-all CRISPR/Cas9 and AAV6-based gene editing strategy to deliver *FOXP3* to its endogenous genomic locus.^36^ Initial protocol optimizations showed 15% and 25% of targeted integration in healthy donor Treg and Teff, respectively. Aside from emulating the wild-type activation/resting kinetics, edited IPEX Treg was able to suppress the proliferation of 40% of wild-type responder Teff compared with nonedited controls. Such promising findings were complemented with *in vivo* experiments in *FOXP3* humanized mice, where gene-corrected IPEX HSPCs persisted in the bone marrow for up to 3 months and were able to differentiate into Treg in the spleen. Further investigation on whether this approach can rescue the *scurfy* mice phenotype, which is an equivalent model for human IPEX, while stabilizing the tight regulation of *FOXP3* during T cell development in the thymus, remains to be sought.

## Challenges of HSPC Gene Editing

Encouraging proof-of-concept data for the precise treatment of PID continue to stem from the research field of genome editing and its innovative therapeutic power. To translate this platform from bench to bedside in a safe and effective way, major challenges need to be addressed during basic and preclinical experiments. Some of these hurdles include:

### Delivery of the editing machinery

One of the crucial processes during *ex vivo* cell engineering is the delivery of the nuclease reagents to the target cells. Apart from the Cas9 nuclease, which is commercially manufactured as purified protein, ZFNs and TALENs must be delivered as either plasmid DNA or mRNA. Electroporation of plasmid DNA is the least used technique due to high toxicity in human primary cells and off-target insertion.^37^ Careful consideration must also be taken when delivering mRNA forms of ZFNs, TALENs, and Cas9 to ensure that premature degradation due to impurities and improper handling, as well as an antiviral response in HSPCs, do not occur.^19,34,38^ These unwanted effects are minimal when delivering the Cas9 coupled to the gRNA in a RNP complex, leading to high editing rates.^39^ Due to their nonintegrative property and high tropism toward HSPCs, viral vectors, such as AAVs and IDLVs, have been extensively adopted not only for nuclease delivery but also to transfer the donor templates for HDR-mediated editing. One of the drawbacks of AAVs is their 4.5 kb cargo capacity, which limits the delivery of large transgenes. In contrast, IDLVs can handle >10 kb transgene cassettes but suffer from low titer, which has a costly implication for large-scale vector production, and non-negligible rates of integration into the genome in a semirandom fashion.^40^ Similarly, lipid and gold-based nanoparticles are considered safer in terms of overall toxicity and immunogenicity profile, but the rate of targeted integration achieved so far in HSPCs is not sufficiently high to fully correct most PIDs.^41^ Currently, AAV6 is considered the genetic tool of choice to deliver the donor template for efficient HSPC gene editing, given its overall low toxicity, minimal rates of random integration and high recombination frequencies even when small homology regions are provided.^39^

### Frequency of HDR-mediated targeted integration

Achieving sustained and higher levels of targeted integration is one of the ultimate goals to completely cure PID by gene editing. However, following the *ex vivo* delivery of the editing reagents, NHEJ is the preferential pathway utilized to correct the DSB in nondividing cells, such as stem cells. To overcome this issue, various groups have implemented strategies to either inhibit NHEJ,^42^ or increase the frequency of HDR,^43^ and have optimized timing and dosage of the editing reagents^19,44^ to enhance knockin efficiency, resulting in targeted integration rates of >60% *in vitro*. Selection of gene-targeted HSCs and/or HSPCs after editing and before transplantation is another potential route to compensate for the low frequency of HDR in stem cells, as it would guarantee the infusion of a virtually pure population of corrected cells. However, further improvements in the selection protocol and cell culture techniques are required before applying this strategy in a clinical setting, since despite an increase in the HDR rate, the yield and the engraftment ability of edited HSCs after cell sorting are dramatically decreased.^39^

### Restoration of therapeutically relevant levels of protein expression

To ensure a therapeutic benefit from the gene editing approach, it is necessary to mediate expression of the corrected gene at curative levels and in the desired cell type upon targeted integration. Site-specific correction or insertion of the donor cassette in its own locus, under the control of its endogenous regulatory regions, secures a regulated protein expression that follows the kinetics observed in wild-type cells and healthy individuals (Fig. 1). However, in the case of site-specific gene insertion, critical regulatory regions contained in introns may be lost when delivering the correct gene in the form of cDNA, highlighting the need of a careful consideration about which part of the mutated locus must be targeted (*e.g.*, exon 1 vs. exon 2) and which genetic elements must be included in the donor template. In this regard, the choice of suitable transcriptional (5’ UTR, Kozak sequences, transcription factor binding sites) and post-transcriptional [3’ UTR, poly(A), WPRE] regulatory sequences in the donor cassette may significantly affect the expression of the corrective protein.^30,33^

### Preservation of the stemness and of the engraftment ability of edited HSPCs *in vivo*

One of the critical aspects of HSPC gene editing that requires thorough evaluation is the capability of corrected stem cells to engraft into the bone marrow *in vivo* while preserving their long-term repopulating potential. While good engraftment rates of up to 60% have been observed following transplant of HSPCs electroporated with Cas9:gRNA RNP into immunodeficient mice, the persistence of edited cells in the hematopoietic tissues decreases significantly within 8–16 weeks after transplant and in serial transplantation experiments.^19,21,30,44^.

The decline in the frequency of corrected cells *in vivo* could be due to the inefficient HDR-mediated editing in quiescent long-term repopulating HSCs, or their inability to self-renew upon their manipulation *in vitro*, including exposure to the editing reagents and culture conditions. Different strategies have been put in place in recent years to maintain and expand the primitive pool of self-renewing HSCs, such as the use of small molecules (UM171, PGE2, and StemRegenin1),^19,20,21,38,39,44^ as well as the optimization of culture conditions and timing of delivery of the editing reagents to HSPCs to preserve their engraftment potential. Some studies have suggested that p53-mediated damage may alter HSC stemness as well as lead to cell cycle arrest, which decreases the frequency of DSB repair by HDR.^38,45^ Although transient p53 inhibition has been sought to eliminate its detrimental effects on stem cells and increase HDR,^45^ the use of such inhibitors in preclinical and clinical settings must be carefully evaluated, considering the essential tumor suppressor function of the p53 pathway. In addition, other groups have uncovered cellular factors, such as the interferon-inducible antiviral factor, which may be responsible for an innate immune response to viruses in cells where the gene editing reagents are delivered by lentivectors; a recent study has shown that this phenomenon could be counteracted by the use of Cyclosporin H.^46^

#### Nuclease specificity

Despite their ability to induce targeted modifications, all the gene editing platforms used so far do not possess perfect specificity and may introduce unintended DSBs at random genomic loci. Off-target modifications introduce permanent genetic mutations that may ultimately lead to cancer. To prevent such risks, comprehensive detection and analysis of the widespread activity of genome editors are vital. Several types of methods to identify off-target indels and chromosomal aberration have been described, each with certain benefits and limitations. Although a gold standard has not been laid out yet to determine which method to apply, initial screening to select the ideal ZFN and TALEN pair or gRNA sequence for a given target and their potential off-target sites are provided through numerous computational algorithms.^47^ Such *in silico* approaches are fast and easy to use, but often fail to give a comprehensive picture of the genome-wide activity of the editing machineries, therefore they should be complemented with unbiased genome-wide detection techniques, including IDLV capture, CIRCLE-seq, GUIDE-seq, DIGEOME-seq, and UDiTaS™.^47^. However, these methods do not always identify all potential off-targets, often show limited sensitivity, and/or are applicable only to nonclinically relevant cell lines, which may exhibit huge variability in terms of nuclease off-target activity compared with the therapeutic target cell type. Recent findings to quantify off-targets *in vivo* are encouraging,^48^ however, efforts are still warranted to develop protocols and reagents suitable for assessing off-target fluctuation in HSPCs before, during, and after transplantation in preclinical and clinical settings. Moreover, the prediction of the impact that unintended genetic modifications may have on cell fitness is not trivial and requires the development of functional readouts of safety that must be tailored to the therapeutic cell type of interest and the editing platform used. In a parallel effort, researchers have tried to improve the specificity of the editing tools to limit their off-target activity. For instance, a key modification for ZFNs and TALENs has been the substitution of amino acid residues in the DNA-binding and cleaving domains, which resulted in enhanced on-target to off-target ratio.^49^ On the other side, the development of chemically modified and shorter gRNAs combined with the use of mutants and high-fidelity Cas9 variants have demonstrated significant improvement in the specificity of the CRISPR/ Cas9 gene editing platforms.^50^ With these modifications, therapeutic gene editing for PID is reaching a crucial milestone, exhibiting improved efficacy and robust safety profiles; the research community must continue designing and improving the editing platforms and apply them in disease-relevant settings, rigorously identifying the frequency, location, and consequence of any potential off-targets.

## Conclusion

During the last few years, tremendous advances have been made in the field of gene editing applied to monogenic disorders. Many proof-of-principle studies applying editing platforms to modify HSPCs, including CRISPR/Cas9 and TALENs, have demonstrated the feasibility of such technologies in correcting the genetic defects underlying the onset of PID. Despite the exciting prospects, no PID clinical trials are currently underway that deploy gene editing technologies. Many research groups are now conducting large-scale preclinical work to assess the long-term safety and efficacy of gene-edited products and to define thresholds of genetic and functional correction needed to reverse the phenotype of each set of PIDs. Fundamental biological concerns remain to be addressed to ensure that significant and unexpected adverse events do not occur. The design of preclinical studies that unequivocally mimics the human clinical trials for a particular PID is therefore desirable, to minimize the translational distance between preclinical and clinical results and ensure that the potential risks for trial participants are negligible. Additionally, direct comparison between gene editing and existing gene therapy platforms or any other treatment option available for a specific PID should be carefully evaluated. This is of paramount importance to assess the applicability and feasibility of gene editing as a curative treatment for such ultra-rare patient populations, especially when taking in account the costs incurred from Good Manufacturing Practice-compliant editing reagents, viral vector production, and the setup of the manufacturing facilities and logistics. Correction of patient HSPCs by means of gene editing remains cumbersome and there are still many challenges facing the field, including the specificity and efficiency of the editing system. Continuous efforts are required to overcome such hurdles, with the final aim of translating gene editing into the next generation of therapeutic tools for severe PID and other blood disorders.
